# Screening pregnant women in a high-risk population with WHO-2013 or NICE diagnostic criteria does not affect the prevalence of gestational diabetes

**DOI:** 10.1038/s41598-021-84918-y

**Published:** 2021-03-10

**Authors:** Mohammed Bashir, Ibrahim Ibrahim, Fatin Eltaher, Stephen Beer, Khaled Baagar, Mahmoud Aboulfotouh, Justin C. Konje, Abdul-Badi Abou-Samra

**Affiliations:** 1 Qatar Metabolic Institute, Endocrine Department, Hamad Medical Corporation, Doha, Qatar; 2 Women Wellness and Research Centre, Hamad Medical Corporation, Doha, Qatar; 3 Sidra Medicine, Qatar Foundation, Doha, Qatar; 4 Al-Wakra Hospital, Hamad Medical Corporation, Doha, Qatar

**Keywords:** Diabetes, Gestational diabetes

## Abstract

There are currently several diagnostic criteria for gestational diabetes (GDM). Both the WHO -2013 and NICE diagnose GDM based on a single step 75 g OGT; however; each uses different glucose thresholds. Previous studies have shown that the prevalence of GDM using the NICE criteria (GDM-N) is lower than that using the WHO-2013 criteria (GDM-W). Qatar has national diabetes in pregnancy program in which all pregnant women undergo OGTT screening using the WHO-2013 criteria. This study aims to define the prevalence of GDM using both criteria in a high-risk population. This retrospective study included 2000 women who underwent a 75 g (OGTT) between Jan 2016 and Apr 2016 and excluded patients with known pre-conception diabetes, multiple pregnancy, and those who did not complete the OGTT. We then classified the women into GDM-W positive, GDM-N positive but GDM-W negative, and normal glucose tolerance (NGT) population. A total of 1481 women (74%) had NGT using the NICE or the WHO-2013 criteria. The number of patients who met both criteria was 279 subjects (14%) with a good agreement (Kappa coefficient 0.67, p < 0.001). The NICE and the WHO-2013 criteria were discordant in 240 subjects (12% of the cohort); 6.7% met the WHO -2013 criteria only and only 5.3% met the NICE criteria. The frequency of pre-eclampsia, pre-term delivery, Caesarean-section, LGA and neonatal ICU admissions were significantly increased in the GDM-W group. However, the GDM-N positive but GDM-W negative had no increased risk of maternal or fetal complications apart from pregnancy-induced hypertension. The WHO-2013 and the NICE criteria classified a similar proportion of pregnant women, 21.5% and 20.1%, respectively, as having GDM; however, they were concordant in only 14% of the cases. Women who are GDM-N positive but GDM-W negative are not at increased risk of maternal and fetal pregnancy complications, except for pregnancy-induced hypertension. As the NICE criteria are more specific to the UK population, we would recommend the use of the WHO-2013 criteria to diagnose GDM in the MENA region and possibly other regions that do not have the same set-up as the UK.

## Introduction

Gestational diabetes mellitus (GDM) is defined as hyperglycaemia first detected during pregnancy that is neither type 1 diabetes mellitus (T1DM) or type 2 diabetes mellitus (T2DM)^1^. Few areas of diabetes care have generated as much debate, controversy, and lack of consensus as GDM. Discussions cover the diagnostic criteria, classification, timing of screening, and method of screening (universal versus selective screening)^1–6^. The HAPO trial (Hyperglycaemia and Adverse Pregnancy Outcomes) followed 25,505 pregnancies from different ethnicities; who underwent 75 g OGTT (Oral Glucose Tolerance Test) between 24- 32 weeks’ gestation^7^. This study showed a continuous association between fasting, 1-h and 2-h blood glucose and the subsequent risk of large for gestational age (LGA), C-section and cord-serum C-peptide^7^.

In 2010 the International Association of Diabetes and Pregnancy Study Groups Recommendations (IADPSG) published new diagnostic criteria for the diagnosis of GDM based on the risks of large for gestational age (LGA)^8^. Using fasting blood glucose (FBG), 1-h, and 2-h OGTT plasma glucose concentrations of 4.5, 7.4, and 6.2 mmol/l, respectively as references; the panel calculated odds ratio of various glucose cut points. The glucose threshold for the diagnosis of GDM was selected based on an odds ratio of 1.75 for LGA compared to the reference glucose levels^8^. In 2013; these new diagnostic thresholds for GDM (FBG ≥ 5.1 mmol/l, 1-h post-OGTT ≥ 10.0 mmol/l or 2-h post-OGTT ≥ 8.5 mmol/l)were adopted by the WHO^1^. The adoption of these new criteria has resulted in an increase in the prevalence of GDM by approximately 20%^9,10^.

﻿ The National Institute for Health and Care Excellence (NICE) recommends against the use of the IADPSG criteria based on health economic modelling using a wide range of glucose thresholds. The model included shoulder dystocia, CS, neonatal jaundice, pre-eclampsia, induction of labour and neonatal intensive care unit admission. Diagnostic thresholds of fasting glucose of ≥ 5.6 mmol/l and a 2 h post 75 g glucose load of ≥ 7.8 mmol/l were felt to be best supported by the health economic analysis^4^. NICE, raised concern about the rise in the prevalence of GDM and the poor health and economic evaluation of the IADPSG diagnostic criteria. Despite being developed for the United Kingdom population, the NICE-GDM criteria are widely used in the MENA-(Middle East and North Africa) region.

Qatar is a growing urban country in the Middle-East with a high prevalence of obesity and type 2 diabetes in the general population^11^. The government has developed a nationwide program in which all women are screened for diabetes in pregnancy using the WHO-2013 criteria^12^. Using the above criteria, the prevalence of newly detected diabetes in pregnancy in Qatar is 24.0%^13^. This high prevalence of GDM in Qatar represents a significant burden on current and future healthcare resources; and increasing psychological stress on pregnant women. Previous studies have reported lower rates of GDM using NICE criteria compared to the WHO-2013 criteria^14–16^. The primary objective of this study was to investigate whether or not NICE criteria is associated with a lower prevalence of GDM compared to the WHO-2013 criteria in this high-risk population. Furthermore, we aimed to examine the impact of the change in the diagnostic criteria on pregnancy outcomes.

## Methods

The details of the universal screening program for diabetes in pregnancy have been described previously^13^. We performed a convenience sampling and included all women who underwent a 75 g OGTT between January 2016 and April 2016 in the Women’s Hospital Hamad Medical Corporation, Doha, Qatar. This is the main maternity hospital in Qatar with an annual birth rate of 16,000–18,000. Subjects were identified using the laboratory database. We excluded all women who did not complete the full two hours OGTT -unless the fasting blood glucose was diagnostic of diabetes in pregnancy; those known to have pre-conception diabetes, and multiple pregnancies. Subjects with FBG ≥ 7.0 mmol/l and/or 2 h BG ≥ 11.1 mmol/l were classified as T2DM and were not included in the analysis of pregnancy outcomes. We defined GDM using the WHO -2013 criteria as (FBG ≥ 5.1 mmol/l, 1-h post OGTT ≥ 10.0 mmol/l or 2-h post OGTT ≥ 8.5 mmol/l); and using the NICE criteria as (FBG ≥ 5.6 mmol/l, or 2-h post OGTT ≥ 7.8 mmol/l).

All GDM patients are managed by nutritional therapy for 1–2 weeks. If 20% or more of the self-monitoring blood glucose readings are above the ADA targets (FBG ≤ 5.3 mmol/L, I-hour post prandial ≤ 7.8 mmol/L or 2 h ≤ 6.7 mmol/L), medical therapy is then added^17,18^. Metformin is the first-line medical therapy unless it is contraindicated, unacceptable to the patient, or is not tolerated. Insulin is used as supplementary to metformin or solely if metformin was not tolerated or could not be used. Obese women with normal glucose tolerance (NGT) do not routinely receive nutritional therapy.

For the maternal and neonatal outcomes, we defined three groups. The first group (GDM-W) fulfilled the WHO-2013 diagnostic criteria, the second group (GDM-N) fulfilled the NICE diagnostic criteria but were negative using the WHO-2013 criteria and the third group tested negative for GDM by both criteria (NGT).

**Figure 1:**
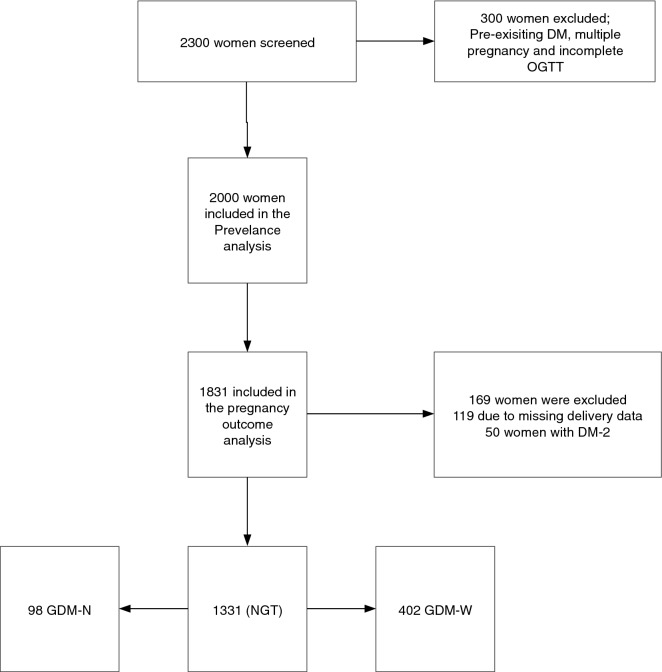
The study flow chart.

All maternal and neonatal data were collected from the electronic medical record. For the sake of the prevalence analysis we included all 2000 women- as shown in Fig. 1. For the pregnancy outcomes we excluded 169 women, 50 with newly detected DM-2 and 119 with missing delivery data. Patient’s ethnicity was classified as Qatari; non-Qatari Arab (residents of the Middle East and North Africa Region); Asian (residents from the India sub-continent and the Philippines) and others. Pre-pregnancy weight was recorded in the first visit based on patient self-report and was entered into the electronic medical records as “pre-pregnancy weight”^13^. If this was not recorded, we used the last recorded weight before conception as pre-pregnancy weight otherwise the weight was considered to be missing. We used the last height recorded before conception or the height recorded in the first antenatal visit to calculate the BMI^13^. Maternal age was calculated as the age of the mother at conception. Large for gestational age (LGA) is defined as birth weight > 90th percentile for gestational age, and small for gestational age (SGA) is defined as birth weight < 10th percentile for gestational, using the locally adapted growth charts. Pre-term delivery is defined as delivery < 37 complete weeks of gestation^13^. Hypertensive disorders in pregnancy were diagnosed based on the American College of Obstetrics and Gynaecology guidelines^19^. The study was approved by the Institutional Review Board of Hamad Medical Corporation. All research was performed in accordance with relevant guidelines/regulations. Due to the nature of the study a waiver of the informed consent was obtained by the Institutional Review Board of Hamad Medical Corporation^13^.

Statistical analysis was performed using STATA 15 software (College Station, TX: Stata Corp LP). Categorical variables were expressed as numbers of percentages (%) and means ± standard deviation were calculated for normally distributed continuous variables. Prevalence was expressed as a percentage (%) with 95% logit confidence interval (CI). Student t-test was used to compare continuous variables, while the Chi-square test was used to compare categorical variables. We examined the agreement rate between the two diagnostic criteria using Cohen’s Kappa coefficient. P value < 0.05 was considered significant.

## Results

Two thousand patients (2000) were included in the study. Of those 754 (37.7%) were Qatari, 705 (35.3%) were non-Qatari Arabs and 446 (22.3%) were Asians and 95 (4.8%) were others. As shown in Table 1, 70.7% of the cohort were either obese or overweight. The non-Qatari Arabs cohort had the highest prevalence of obesity, (50%) followed by the Qataris (46%).

**Table 1:** Baseline characteristics of women.

	Qatari	Arab	Asian	Other	Total
Age in years* (mean ± SD)	29.8 ± 5.9	30.3 ± 5.6	30.4 ± 5.0	32.0 ± 5.8	30.2 ± 5.6
BMI (kg/m^2^)** (mean ± SD)	30.0 ± 6.8	30.4 ± 6.2	27.5 ± 5.3	28.5 ± 6.3	29.5 ± 6.4
BMI categories** n (%)					
Underweight (< 18)	2 (0.3%)	2 (0.3%)	1 (0.2%)	0 (0.0%)	5 (0.3%)
Normal (18–24.9)	162 (21.5%)	131 (18.6%)	142 (31.8%)	29 (30.5%)	464 (23.2%)
Overweight (25–29.9)	206 (27.3%)	214 (30.4%)	170 (38.1%)	30 (31.6%)	620 (31.0%)
Obese 1 (30–34.9)	215 (28.5%)	178 (25.2%)	72 (16.1%)	18 (18.9%)	483 (24.1%)
Obese 2 (35–39.9)	92 (12.2%)	105 (14.9%)	28 (6.3%)	8 (8.4%)	233 (11.7%)
Obese 3(≥ 40)	40 (5.3%)	44 (6.2%)	9 (2.0%)	5 (5.3%)	98 (4.9%)
Total	754 (100%)	705 (100%)	446 (100%)	95 (100%)	2000 (100%)

*Age has 0.05% missing observations.

**BMI and weight have 4.9% missing observations.

The prevalence of GDM based on the WHO-2013 criteria was 21.5% (95% CI 19.7–23.3), whereas the prevalence was 20.1% (95% CI 18.4%, 21.9%) when applying the NICE criteria (Table 2).As shown in Table 3, 26% of the women were identified as having GDM using either the WHO-2013 or the NICE criteria or both. Both criteria showed good agreement on the diagnosis of GDM in 279 out of 2000 women (14.0%); Kappa coefficient 0.67; p < 0.001.

**Table 2:** The prevalence of newly detected diabetes in pregnancy using the WHO-2013 and the NICE criteria.

	GDM-WHO-2013		GDM-NICE	
	Number of Women	Prevalence (95% CI)	Number of Women	Prevalence (95% CI)
DM	50	2.5% (1.9, 3.3)	50	2.5% (1.9, 3.3)
GDM	429	21.5% (19.7, 23.3)	401	20.1% (18.4%, 21.9%)
Total	479	24.0% (22.1, 25.9)	451	22.6% (20.8, 24.4%)

**Table 3:** The classification of the study population according to the OGTT results and GDM diagnostic criteria.

	Number	%
GDM based on both WHO-2013 and NICE criteria	279	14.0
GDM based on WHO-2013 criteria but not meeting NICE criteria	134	6.7
GDM based on NICE criteria but not meeting WHO -2013 criteria	106	5.3
Type 2 diabetes	50	2.5
NGT with both criteria	1431	71.5
Total	2000	100

Kappa score 0.67; p < 0.001.

For pregnancy outcomes, we included only women in whom pregnancy outcomes were available. Table 4 shows that women in both the GDM-W and the GDM-N had high BMI compared to the (NGT) (31.7 ± 6.3 and 30.2 ± 5.7 vs 28.7 ± 6.2; p = 0.001 and 0.033 respectively). Both the GDM-W and the GDM-N had higher rates of PIH compared to the NGT (4.5% and 5.1% vs 2.5%; p = 0.007 and 0.048 respectively).

**Table 4:** The characteristics and pregnancy outcomes.

	GDM-N (98)	GDM-W (402)	NGT (1331)	P value	
				NICE Vs Normal	WHO Vs Normal
Age (years)	29.0 ± 7.0	31.7 ± 5.6	30 ± 5.7	0.095	0.974
BMI (kg/m^2^)^†^	30.2 ± 5.7	31.7 ± 6.3	28.7 ± 6.2	0.033	0.001
Ethnicity	Qatari (43.9%)	Qatari (43.3%)	Qatari (37.2%)	0.4	0.097
	Arab (34.7%)	Arab (30.4%)	Arab (36.2%)		
	Asian (19.4%)	Asian (22.1%)	Asian (21.7%)		
	Others (2.0%)	Others (4.2%)	Others (4.9%)		
PIH	5 (5.1%)	18 (4.5%)	26 (2.0%)	0.048	0.007
Pre-eclampsia	5 (5.1%)	11 (2.8%)	30 (2.3%)	0.09	0.637
Pre-term delivery	13 (13.5%)	69 (17.2%)	145 (10.9%)	0.432	0.001
Induction of labour	15 (15.3%)	61 (15.3%)	136 (10.2%)	0.112	0.005
CS	41 (41.7%)	168 (41.8%)	462 (34.7%)	0.151	0.010
**Neonatal outcome**				0.013	0.102
Live birth	95 (96.9%)	396 (98.5%)	1322 (99.3%)		
Still birth	3 (3.1%)	5 (1.24%)	9 (0.7%)		
Miscarriage	0%	1 (0.3%)	0%		
**Weight percentile**				0.744	< 0.01
AGA	76 (79.2%)	298 (75.2%)	1044 (79.0%)		
LGA	8 (8.3%)	55 (13.8%)	88 (6.7%)		
SGA	12 (12.5%)	43 (11.0%)	190 (14.4%)		
NICU	4 (4.1%)	58 (14.7%)	110 (8.3%)	0.140	< 0.01
Resp distress	2 (2.1%)	32 (8.1%)	53 (4.9%)	0.200	0.017
Neo hypo	1 (1.0%)	45 (11.3%)	38 (2.9%)	0.284	< 0.01
Shoulder dystocia	0 (0%)	2 (0.51%)	4 (0.3%)	0.587	0.555

## Discussion

To our knowledge, this the first study to compare the prevalence of GDM using two different diagnostic criteria, in a country from the MENA area (the Middle East and North Africa), which has the second-highest prevalence of hyperglycaemia in pregnancy^20^. In this study, the two set of GDM criteria showed a good agreement and were concordant in 67% and 72% of WHO-2013 and NICE identified subjects, respectively, the remaining 33% and 28% of WHO-2013 and NICE identified subjects, respectively, were normoglycemic by the other set of criteria. Thus about 30% of subjects identified by one set of GDM criteria can be considered normal by the other set. Despite this fact, GDM prevalence was not much different between the WHO-2013 or the NICE criteria, 21.5% and 20.1% respectively. The use of different criteria within the same population resulted in a re-classification of 12% of the population as either normal or GDM. Apart from an increase in the rates of pre-eclampsia (PET), women who were detected with GDM based on NICE alone has similar fetal and maternal outcomes compared to the general population. It should be noted that the rates of still birth were extremely low in the whole cohort [17/1831 (0.92%)], and hence difference between the groups is subjected to chance alone. Indeed, two of the three cases of the stillbirth in the GDM-N group were due to multiple congenital malformation, and the third case was due to placental thrombosis secondary to protein S deficiency.

Our findings are in keeping with one study from Vietnam that reported a similar prevalence of GDM with both the NICE and WHO-2013 criteria; 24.2% and 22.8%^21^. Other studies have consistently reported a lower prevalence of GDM with NICE criteria than with the WHO-2013 criteria. A study of 554 women from South Africa reported a GDM-N prevalence of 17.0% compared to a GDM-W prevalence of 25.1%^15^. A study from Croatia reported a GDM-N prevalence of 17.8% and a GDM-W prevalence of 23.1%^14^. A study of 680 women from India and reported a prevalence of GDM-N of 11.6% and GDM-W of 25.1%^16^. A study from Canada reported a GDM-N prevalence of 18% and a GDM-W prevalence of 53%^22^. A Finnish study that included 4033 women reported a GDM-N prevalence of 13% and a GDM-W prevalence of 31%^23^.

In terms of pregnancy outcomes, our data showed that women who are classified as GDM W-negative and GDM-N positive were not at increased risk of pregnancy complications- apart from PET. This is in keeping with studies from both Finland and India that showed that women who are GDM W-negative and GDM-N positive were not at increased risk of pregnancy complications- compared to the background population^16,23^. On the other hand, a Canadian study showed that the NICE criteria didn’t identify women with an increased risk of pregnancy complications^22^. Furthermore, a UK-based study showed that women who are GDM W-positive and GDM-N negative had similar risk of complications compared to the GDM-N^24^.

Blood glucose level is a continuum; cut-off points are essentially arbitrary. There is no dispute that hyperglycaemia in pregnancy carries increased maternal and fetal risks; however, the dispute is which one(s) should define the glucose thresholds for GDM. It is perhaps more critical to have one single definition of GDM within the same health care country. As shown in this study, the use of multiple diagnostic criteria resulted in re-classification of the population with no impact on the prevalence of GDM. Furthermore, the use of multiple diagnostic criteria within the same country can result in extreme differences between centres. For example, two studies from Saudia Arabia reported a GDM prevalence of 19.6% using the two steps Carpenter-Coustan Criteria and 50.1% using the WHO-2013 criteria^25,26^. The absence of a single definition of GDM within the same country makes it more challenging to allocate resources and monitor the adequacy of medical management in reducing the risks of pregnancy complications. Furthermore, pregnancy provides an ideal opportunity to engage women in programs to improve future health. Without a unified definition for GDM it will not be feasible to appropriately define the post-natal progression rates to type 2 diabetes mellitus in women with GDM; an issue that can hinder efforts to develop national diabetes prevention programs.

Since there are no head- to head trials to compare the WHO-2013 and the NICE, it is quite critical to understand the differences between the two diagnostic criteria. The WHO-2013 criteria is based on the HAPO trial which was a large—multi-ethnic-prospective study^7^. The NICE guidelines was based on composite adverse pregnancy outcomes with consideration to health benefits and resources uses that are specific to the United Kingdom^4^. As shown above, studies from various countries showed that the NICE criteria do not detect a high-risk group. Hence, we can argue that the NICE- GDM criteria are not applicable globally. However, countries might use the NICE-methodology to define the glucose thresholds based on their own adverse pregnancy complications and economic considerations- rather than using the NICE—glucose thresholds.

## Conclusion

In a high-risk population, the use of two different diagnostic criteria did not impact the overall prevalence of GDM but resulted in a re-classification of 12.8% of the population. We, therefore, recommend the use of a single set of criteria within the same country. As the NICE criteria are more specific to the UK population, we would recommend the use of the WHO-2013 criteria to diagnose GDM in the MENA region and possibly other regions that do not have the same set-up as the UK.
